# AAV2-associated liver injury across infection and gene therapy: converging genomic signals

**DOI:** 10.3389/fimmu.2026.1808458

**Published:** 2026-04-30

**Authors:** Yuanzhi Chen, Qing Zhu, Na Yuan, Dian Jin, Zhenzhen Zhu, Fentian Chen

**Affiliations:** 1 School of Medicine, Huaqiao University, Xiamen, China; 2 Department of General Surgery, Xiamen Chang Gung Hospital, Hua Qiao University, Xiamen, China; 3 State Key Laboratory of Vaccines for Infectious Diseases, Xiang An Biomedicine Laboratory, School of Public Health, Xiamen University, Xiamen, China

**Keywords:** AAV2, gene therapy, liver injury, pediatric hepatitis, viral co-infection

## Introduction

Adeno-associated virus (AAV) vectors are widely regarded as safe platforms for *in vivo* gene delivery. Nonetheless, rare but severe cases of hepatotoxicity following high-dose systemic AAV gene therapy continue to raise unresolved mechanistic questions. In a recent Brief Communication, Buddle et al. reported extensive disruption and concatemerization of AAV vector genomes, contamination with manufacturing plasmid sequences, and detection of human betaherpesvirus 6B (HHV-6B) in the liver of a child who developed severe hepatitis following onasemnogene abeparvovec therapy (1). These findings merit broader contextualization in light of earlier genomic investigations of unexplained pediatric hepatitis that independently implicated AAV2. During the global outbreak of acute severe hepatitis of unknown etiology in children reported from 2022 onwards, multiple studies identified striking enrichment of AAV2 DNA in blood and liver samples from affected cases compared with controls (2–4). Importantly, these investigations consistently showed that AAV2 was detected at high copy numbers, frequently accompanied by low-level adenovirus or HHV-6, whereas adenovirus alone did not fully account for disease severity. Commentaries and analyses across multiple journals converge on the view that AAV2 detection in pediatric acute hepatitis likely reflects a multifactorial process involving helper-virus co-infection, host genetic susceptibility and immune-mediated liver injury rather than a single viral cause, while emphasizing that causality remained unproven (5–8).

## AAV2 detection in pediatric hepatitis

Across three independent cohorts, Morfopoulou et al. demonstrated widespread detection of AAV2 in children with acute hepatitis, often alongside potential helper viruses (2). Ho et al. reported an association between AAV2 infection, specific HLA class II alleles, and severe liver inflammation, suggesting host genetic susceptibility (3). Baker et al. further observed that co-detection of AAV2 and adenovirus correlated with more severe disease than adenovirus alone (4). Collectively, these studies challenged the prevailing assumption that wild-type AAV infection is uniformly benign in immunocompetent hosts. Subsequent epidemiological investigations have reinforced this association. A national investigation from Ireland reported frequent detection of AAV2 in children with acute hepatitis of unknown origin, often accompanied by adenovirus or herpesvirus co-infection (9). Additional cohort analyses indicate that AAV2-associated pediatric hepatitis may occur sporadically outside the 2022 outbreak, consistent with a multi-hit model involving AAV2 infection, helper viruses and host immune responses leading to immune-mediated liver injury. (10, 11). Mechanistic studies have also provided complementary insights. Spatial immune profiling of liver biopsies from affected children revealed CD8^+^ T-cell–dominated inflammatory microenvironments, consistent with immune-mediated liver injury rather than direct viral cytopathic effects (12). Consistent with these observations, recent reviews propose that acute hepatitis of unknown origin in children likely reflects a complex interplay between adenovirus infection, AAV2 co-infection and host genetic susceptibility rather than a single pathogenic agent (13).

## AAV-derived DNA complexity in gene therapy–associated hepatotoxicity

The Brief Report by Buddle et al. introduces a parallel line of evidence from the gene therapy setting (1). Using long- and short-read metagenomic sequencing of liver tissue, the authors identified complex concatemeric AAV-derived DNA structures, extensive rearrangements involving vector genomes and manufacturing plasmid sequences, and numerous vector–human junctions. HHV-6B DNA was also detected, although without evidence of active viral replication. Although derived from a single case and therefore not necessarily representative of broader AAV biology, these findings demonstrate that complex AAV-derived DNA species can persist in human liver tissue under certain conditions.

## Discussion

Taken together, the pediatric hepatitis outbreak studies (2–4, 9–14) and the post–gene therapy hepatitis case (1) suggest convergent biological themes. In both contexts, AAV2 or AAV-derived genomes are present at unexpectedly high levels, often accompanied by potential helper viruses, and associated with liver inflammation. These observations raise the possibility that under certain conditions—such as high viral or vector genome burden, permissive co-infections, or host genetic susceptibility—AAV-derived DNA structures may contribute to hepatic immune activation. Mechanistically, wild-type AAV2 requires helper viruses for productive replication, and experimental systems have shown that herpesvirus co-infection can facilitate rolling-circle or rolling-hairpin amplification of AAV genomes. Aberrant replication intermediates, concatemeric DNA, or integration events may expose immunostimulatory DNA motifs or generate atypical transcriptional products capable of triggering innate or adaptive immune responses. In the liver, a highly immunologically active organ, such processes could plausibly precipitate hepatitis in susceptible individuals. In addition, intrahepatic heterogeneity may further influence disease expression. The liver comprises diverse hepatocyte zonation patterns and multiple non-parenchymal cell populations, each with distinct immune and metabolic profiles. Such microenvironmental variability may help explain why similar AAV genome burdens could result in divergent clinical outcomes both between patients and within different regions of the same liver.

Beyond mechanistic considerations, these convergent observations raise potential translational implications. Complementary serological analyses have identified conserved AAV2-specific antibody signatures enriched in affected children, consistent with these AAV2 infections (14). If high AAV2 genome burden contributes to hepatic immune activation, therapeutic neutralization of AAV2 may warrant exploration. Neutralizing antibodies are well established as protective or disease-modifying agents in other viral infections, and capsid-directed antibodies are already recognized as major determinants of vector biology in gene therapy. Whether AAV2-specific monoclonal antibodies could meaningfully reduce viral load or inflammatory signaling in the context of AAV-associated hepatitis remains entirely speculative and would require rigorous preclinical validation. In parallel, host determinants of AAV tropism merit renewed attention. Although AAV2 has historically been regarded as broadly hepatotropic, the molecular basis of its interaction with hepatocytes remains incompletely defined. Recent work identifying a previously unrecognized cellular receptor for AAV8 underscores that AAV serotype–specific entry mechanisms continue to be uncovered (15). This finding raises the possibility that hepatocytes, or specific hepatic cell subsets, may express yet-unidentified receptors or co-receptors preferentially facilitating AAV2 entry or persistence. Identification of an AAV2-specific receptor could not only illuminate susceptibility to AAV-associated hepatitis but also inform both vector engineering and therapeutic intervention strategies. In this context, the discovery of an AAV2 receptor may be a matter of timing rather than principle.

Importantly, these considerations extend beyond natural infection. Although recombinant AAV vectors used in gene therapy are replication-defective, the presence of rep/cap fragments, helper-derived sequences, or contaminating plasmid DNA—as observed by Buddle et al. (1*)*—raises the possibility that similar DNA processing pathways may be engaged *in vivo*. Whether these complex DNA species are merely epiphenomena or directly pathogenic remains unknown. However, their detection in a case of severe liver injury underscores the need for systematic investigation.We propose that future studies of AAV gene therapy–associated hepatotoxicity integrate unbiased genomic profiling of vector-derived DNA structures in target tissues, alongside immune phenotyping and assessment of co-viral status. Likewise, mechanistic insights from pediatric hepatitis associated with AAV2 infection (2–4) may inform safety evaluation of therapeutic vectors, particularly as increasingly high systemic doses are deployed.

Clarifying whether complex AAV-derived DNA structures represent a shared pathway linking infection-associated hepatitis and gene therapy–related liver injury will be critical for refining both clinical management and vector manufacturing strategies. At minimum, the accumulating evidence suggests that AAV biology in the human liver is more context-dependent than previously appreciated, with implications for both infectious disease and translational gene therapy. Future studies should prioritize integrated genomic, virological, and immunological profiling of liver tissue in both infection-associated and gene therapy–associated contexts. Longitudinal sampling, single-cell analyses, and careful assessment of co-viral dynamics will be critical to determine whether complex AAV-derived DNA structures are drivers or bystanders in hepatic inflammation. Such work will be essential for refining patient stratification, vector design, and potential interventional strategies.
